# Parasympathetic activity and total fibrotic kidney in autosomal-dominant polycystic kidney disease patients: a pilot study

**DOI:** 10.1007/s11255-023-03551-y

**Published:** 2023-04-12

**Authors:** Silvia Lai, Adolfo Marco Perrotta, Valeria Panebianco, Sandro Mazzaferro, Paolo Menè, Chiara Pellicano, Francesca Tinti, Maurizio Muscaritoli, Rosario Cianci, Antonietta Gigante

**Affiliations:** 1 Department of Translational and Precision Medicine, Nephrology Unit, Sapienza University of Rome, Viale Dell’Università 37, 00185 Rome, Italy; 2 Department of Radiological, Oncological and Anatomo-Pathological Sciences, Sapienza University of Rome, Rome, Italy; 3 Department of Clinical Sciences, Division of Nephrology, University of Rome La Sapienza, Sant’Andrea University Hospital, Rome, Italy

**Keywords:** Autosomal-dominant polycystic kidney disease, Renin–angiotensin–aldosterone system, Heart rate variability, Total kidney volume, Total fibrotic volume

## Abstract

**Purpose:**

Renin–angiotensin system hyperactivation in autosomal-dominant polycystic kidney disease (ADPKD) patients leads to early hypertension. Cystic enlargement probably causes parenchymal hypoxia, renin secretion, and endothelial dysfunction. Sympathetic and parasympathetic balance is altered in this condition, especially during the night, also affecting blood pressure circadian rhythm. Aim of this study was to evaluate sympathetic/parasympathetic balance using heart rate variability (HRV) parameters and find a correlation between HRV and renal damage progression, as total kidney volume enlargement, in ADPKD patients.

**Methods:**

Sixteen adult ADPKD patients were enrolled in the study. Eleven patients (68.8%) were male, and the median age was 42 years (IQR 36–47.5). HRV parameters were calculated using 24 h-ECG Holter. A kidney magnetic resonance imaging (MRI) scan 3 Tesla was performed to evaluate total kidney volume (TKV) and total fibrotic volume (TFV).

**Results:**

A statistically significant positive linear correlation was observed between length of kidneys and frequency domain parameters as low frequency (LF) (*r* = 0.595, *p* < 0.05) and LFday (*r* = 0.587, *p* < 0.05). Moreover, a statistically significant positive linear correlation exists between high frequency (HF) and TFV (*r* = 0.804, *p* < 0.01) or height-adjusted (ha) TFV (*r* = 0.801, *p* < 0.01). Finally, we found a statistically significant positive linear correlation between HFnight and TKV (*r* = 0.608, *p* < 0.05), ha-TKV (*r* = 0.685, *p* < 0.01), TFV (*r* = 0.594, *p* < 0.05), and ha-TFV (*r* = 0.615, *p* < 0.05).

**Conclusion:**

We suppose that the increase in TKV and TFV could lead to a parasympathetic tone hyperactivation, probably in response to hypoxic stress and vasoconstriction due to cystic enlargement.

## Introduction

Autosomal-dominant polycystic kidney disease (ADPKD) is an inherited monogenic disease, with a prevalence ranging from 1 in 543 to 1 in 4000 [1]. It is characterized by the growth of cystic in kidney, due to a mutation in polycystin 1 and 2, expressed on primary cilium and much more rarely by other recently identified genes as GANAB [2], PMM2 [3], DNAJB11, ALG9, and IFT140 [4]. Cysts growth leads to a parenchymal overthrow causing renal function impairment. It is well known that cardiovascular risk is higher in these patients, regardless their renal function [5, 6]. In particular, renin–angiotensin–aldosterone system (RAAS) is hyperactivated rapidly in the absence of clinically hypertension and the loss of renal function. This evidence could be explained by the effects of cystic enlargement on renal vessels with parenchymal ischemia. Altered intrarenal hemodynamic causes endothelial dysfunction, impairment in nitric oxide (NO) production, and hyperactivation of the sympathetic nervous system (SNS), mainly during the night [7]. The blood pressure circadian rhythm is also lost in this condition. For these reasons, an early detection of these alteration could be useful to address an adequate therapy. Sympathetic/parasympathetic balance could be a very early marker to evaluate. Heart rate variability (HRV) analysis based on 24-h ECG Holter [8] with specific parameters on time and frequency domain is a simple and non-invasive test to assess the risk of cardiovascular events due to sympathetic/parasympathetic imbalance [9].

With this background, the aim of this pilot study was to assess in ADPKD patients the sympathetic/parasympathetic balance using HRV parameters and correlate it with renal damage progression, as total kidney volume enlargement.

## Materials and methods

We enrolled ADPKD patients in this study. DNA samples were analyzed in all patients for PKD genetic diagnosis and mutation in the PKD1 gene was found. Fifteen patients had a family history of ADPKD, except one patient who had a de novo mutation. No patient was in dialysis treatment. Patients with diabetes were excluded a priori*.*

Inclusion criteria were rapidly progressive disease (as kidney function loss > 5 ml/min/year), CKD ≥ stage G3b corresponding to estimated glomerular filtration rate (eGFR) 30–44 ml/min/1.73 m^2^ (Kidney Disease Outcome Quality Initiative (KDOQI) staging system), absence of cardiovascular events, blood pressure (BP) values in normal range under control with one of the following drugs: angiotensin-converting enzyme inhibitors (ACEi), angiotensin II receptor blockers (ARBs), and calcium channel blockers (CCB). The tests were performed before taking tolvaptan therapy. No patient was on beta-blocker therapy, 6 patients were on ACEi, 6 patients were on ARBs, and 11 were on CCB. No patient was on erythropoietin treatment for anemia. At the time of enrollment, medical records, physical assessment, and laboratory data were recorded in all patients. The study protocol was approved by the Local Clinical Research Ethics Committee. The study conforms to the principles outlined in the Declaration of Helsinki and a written consent by each patient enrolled was obtained.

### Magnetic resonance imaging

All patients were subjected to a novel MRI protocol of advanced imaging with magnet 3Tesla (T) (Discovery MR 750, 3 T, GE Healthcare) after positioning of the surface coil 32 channels. Kidney MRI scan 3 T, with and without contrast, was performed to evaluate total kidney volume (TKV) and total fibrotic volume (TFV). The acquisition protocol included morphological sequences, single shot T2-weighted (SSFS) (TR 850 ms, TE 105 ms; Flip Angle 90°; FoV 320 × 320; Matrix 320 × 224) acquired on axial, sagittal, and coronal planes and Gradient Echo (GRE) T1-weighted (TR 5 ms; TE 1 ms; Flip Angle 15°; FoV 420 × 420; 288 × 192 matrix). For the evaluation of parenchymal perfusion, ultrafast GRE T1-weighted sequences were used, acquired in the coronal plane (TR 2 mS; TE 1 mS; Flip Angle 13°; Thickness 200 mm; FoV 300 × 300 mm, matrix 192 × 138) during administration dynamic of i.v. contrast (gadobutrol 1 mmoL/ml, Gadovist, Bayern, Germany) using a perfusion technique, with high temporal resolution of 4 s, for a total duration of about 8 min. The end of the dynamic sequence was made to coincide with the start of the administration of contrast material i.v.19. The evaluation of total perfusion volume (TPV) and TFV results from perfusional MRI after a qualitative and quantitative approach. Each parameter resulted from a post-processed slice by slice renal segmentation, respectively, in early arterial phase (first minute of perfusion) and late perfusional phase (eighth minute of perfusion). Segmentation was guided using colorimetric maps. After segmentation, software Workstation vers. 4.6 was used for three-dimensional (3D) volume rendering reconstruction, which resulted in semiquantitative estimation of parenchymal perfonded tissue and fibrotic areas. These perfusional parameters give indication of functional parenchymal areas. Perfusion volume indicates kidney areas where blood flow is preserved, which is an indirect sign of normal functional parenchyma. Fibrotic areas indicate parenchymal areas that underwent fibrotical substitution and consequent loss of function [10].

### Heart rate variability

Autonomic nervous activity was evaluated by HRV analysis during 24-h ECG recording following the recommendations of the Taskforce of the European Society of Cardiology and the North American Society of Pacing and Electrophysiology [11]. The time of registration was divided into two periods: day (d) (7 a.m. to 12 p.m.) and night (n) (12 p.m. to 7 a.m.). In the time domain, the standard deviation of normal-to-normal RR intervals (SDNN) (ms) and the square root of the mean of the sum of the squares of differences between adjacent NN intervals (RMSSD), representing global sympathetic and the parasympathetic system, respectively, were evaluated. Total power in the frequency domain range (0–0.40 Hz) was divided into low frequency (LF: 0.04–0.15 Hz, modulated mainly by sympathetic system) and high frequency (HF: 0.15–0.40 Hz, modulated by parasympathetic system). The power of LF and HF components was considered in normalized units (nu). LF/HF rate is sympathovagal balance. Data analyses were performed with software Del Mar Avionics Accuplus 363, Irvine California, USA.

### Statistical analysis

Data management and analysis were performed using IBM^®^ SPSS^®^ Statistics 26 for Windows^®^ software (IBM Corporation, Armonk, N.Y., USA). The normality of the variables was tested using the Shapiro–Wilk method for normal distributions. All continuous variables were expressed as median and interquartile range (IQR) and categorical variables were expressed as absolute frequencies and percentages. Spearman or Pearson tests were used for bivariate correlations, as appropriate. A probability value of *p* < 0.05 was considered statistically significant.

## Results

Sixteen adult ADPKD patients were enrolled in this study. Eleven patients (68.8%) were male, and the median age was 42 years (IQR 36–47.5). Median serum creatinine was 1.38 mg/dl (IQR 1.2–1.61), and median eGFR was 58.1 ml/min (IQR 52–60). Median renal resistive index (RRI) was 0.65 (IQR 0.61–0.67). Table 1 shows the anthropometric and clinical features of patients enrolled.

**Table 1:** Demographic and clinical characteristics of enrolled patients

Age, years, median and IQR	42 (36–47.5)
Female/male, *n* (%)	5 (31.3)/11 (68.8)
BMI, Kg/m^2^, median and IQR	24.59 (21.02–25.26)
SBP/DBP, mmHg, median and IQR	120 (110–132.5)/80 (70–85)
Azotemia, mg/dl, median and IQR	50.5 (47.1–57)
Creatinine, mg/dl, median and IQR	1.38 (1.2–1.61)
eGFR, ml/min, median and IQR	58.1 (52–60)
CRP, mg/l, median and IQR	4 (4–8)
NLR, median and IQR	1.65 (1.37–1.98)
Uric acid, mg/dl, median and IQR	6.25 (5–6.9)
Hemoglobin, g/dl, median and IQR	12.8 (12.3–14.2)
Glycemia, mg/dl, median and IQR	82.5 (79–91)
Cholesterol, mg/dl, median and IQR	206 (163–225)
HDL, mg/dl, median and IQR	55 (46–67)
LDL, mg/dl, median and IQR	111.2 (103.4–142.6)
Triglycerides, mg/dl, median and IQR	120 (97–137)
Albumin, g/dl, median and IQR	4.15 (3.8–4.2)
AST, IU/l, median and IQR	21.5 (17–26)
ALT, IU/l, median and IQR	17.5 (12–26)
GGT, IU/l, median and IQR	18 (14–20)
CPK, U/l, median and IQR	144 (119–160)
Na^+^, mmol/l, median and IQR	141 (140–143)
K^+^, mmol/l, median and IQR	4.25 (4.1–4.7)
Cl^−^, mmol/l, median and IQR	105 (102.5–106)
Ca^2+^, mg/dl, median and IQR	9.8 (9.5–10.2)
P^−^, mg/dl, median and IQR	3.4 (3.2–4)
25-OH-vitD, ng/ml, median and IQR	21.7 (16–34.3)
iPTH, pg/ml, median and IQR	61.5 (41–75)
IMT, mm, median and IQR	0.7 (0.67–0.8)
RRI, median and IQR	0.65 (0.61–0.67)

*BMI* body mass index, *SBP* systolic blood pressure, *DBP* diastolic blood pressure, *eGFR* estimated glomerular filtration rate, *CRP* C-reactive protein, *NLR* neutrophil-lymphocytic ratio, *HDL* high-density lipoprotein, *LDL* low-density lipoprotein, *AST* aspartate aminotransferase, *ALT* alanine aminotransferase, *GGT* gamma GT, *CPK* creatine kinase, *iPTH* parathyroid hormone, *IMT* intimal media thickness, *RRI* renal resistive index, *IQR* interquartile range

MRI of the kidneys showed a median TKV of 2102 ml (IQR 1372.5–3155.3) and a median TFV of 298.51 cm^3^ (IQR 177–352.9); median length was 17.97 cm (IQR 16.41–21.39) and median stiffness was 22.13 kPa (IQR 15.8–30). These findings are summarized in Table 2.

**Table 2:** Kidney’s magnetic resonance imaging (MRI) parameters in enrolled patients

TKV, ml, median and IQR	2102 (1372.5–3155.3)
hA-TKV, ml/m, median and IQR	1312.2 (968.7–1783.9)
TFV, cm^3^, median and IQR	298.51 (177–352.9)
hA-TFV, cm^3^/m, median and IQR	182.5 (112–192.84)
RV, cm^3^, median and IQR	556.02 (448.94–790.62)
Length, cm, median and IQR	17.97 (16.41–21.39)
Stiffness, kPa, median and IQR	22.13 (15.8–30)

*TKV* total kidney volume, *hA* height-adjusted, *TFV* total fibrotic volume, *RV* residual volume, *IQR* interquartile range

Median heart rate (HR) was 75.2 bpm (IQR 67.5–82.25) and median of corrected QT interval (QTc) registered was 398 ms (IQR 384–425). Moreover, median SDNN was 127.85 ms (IQR 113.9–168.25) and median RMSSD was 41.35 ms (IQR 27.2–50.5). Table 3 shows HRV parameters during 24-h ECG recording.

**Table 3:** Heart rate variability (HRV) parameters during 24-h ECG recording

VLF, Hz, median and IQR	706 (463–1682)
LF/HF, median and IQR	2.45 (2–2.6)
LF nu, median and IQR	58.85 (55–66.9)
HF nu, median and IQR	32.5 (29.05–36.5)
LFd, Hz, median and IQR	672 (388–1596)
HFd, Hz, median and IQR	411 (245–749)
LF/HFd, median and IQR	2.55 (2.2–2.7)
LFd nu, median and IQR	59.35 (56.5–66.75)
HFd nu, median and IQR	32 (29.5–40)
LFn, Hz, median and IQR	900 (618.5–1723.5)
HFn, Hz, median and IQR	563 (357.5–936.5)
LF/HFn, median and IQR	2.1 (1.85–2.55)
LFn nu, median and IQR	58.5 (52.55–64.15)
HFn nu, median and IQR	34.5 (31.5–39.75)
SDNN, ms, median and IQR	127.85 (113.9–168.25)
RMSSD, ms, median and IQR	41.35 (27.2–50.5)
HR, bpm, median and IQR	75.2 (67.5–82.25)
QTc, ms, median and IQR	398 (384–425)

*VLF* very low frequencies, *IQR* interquartile range, *LF* low frequencies, *HF* high frequencies, *d* day, *n* night, *nu* normal unit, *SDNN* standard deviation of normal-to-normal RR intervals, *RMSSD* square root of the mean of the sum of the squares of differences between adjacent NN intervals, *HR* heart rate, *QTc* corrected QT interval

**Fig. 1:**
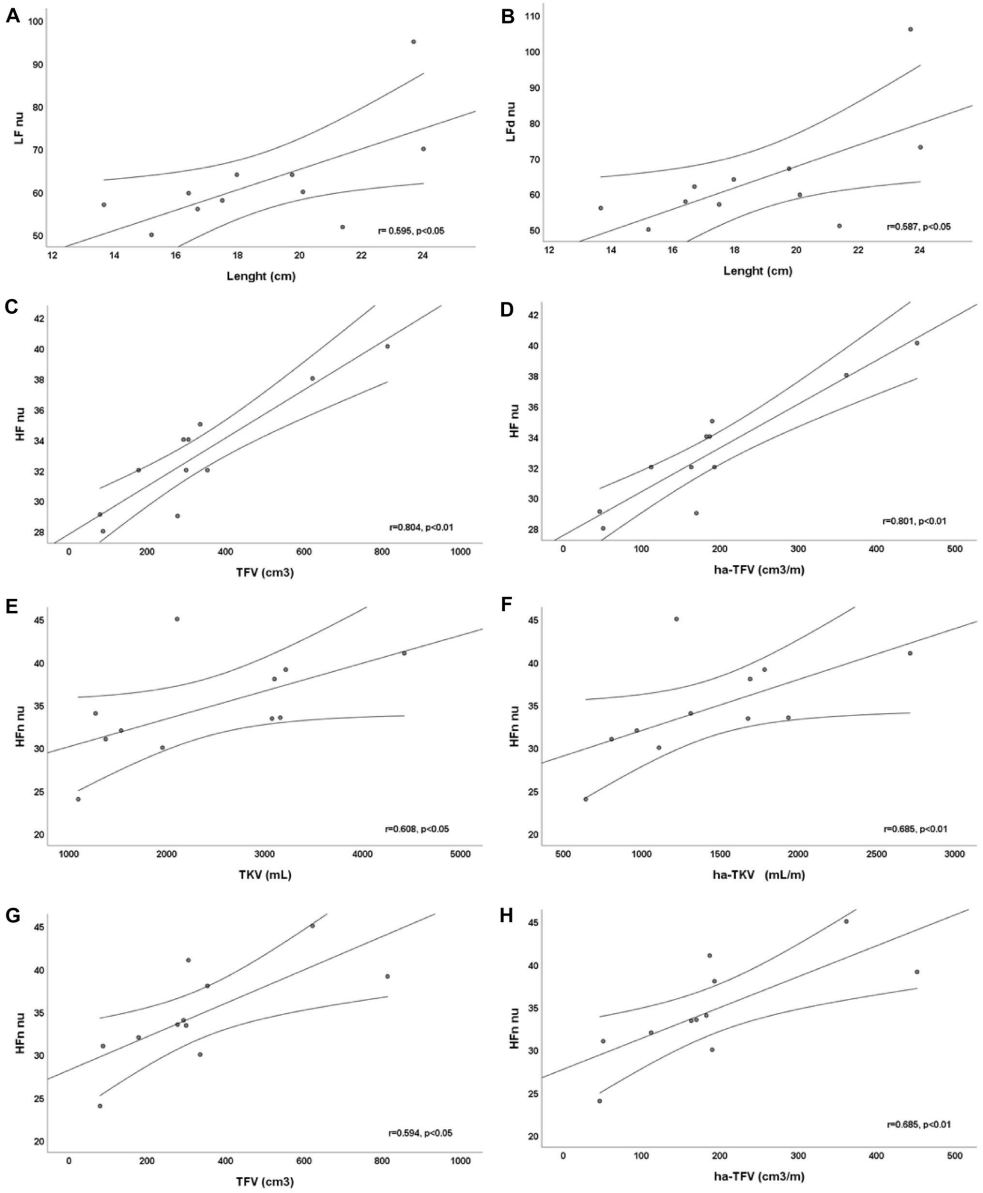
Linear correlation between kidney’s magnetic resonance imaging (MRI) parameters and heart rate variability (HRV) parameters during 24-h ECG recording. *LF* low frequencies, *HF* high frequencies, *d* day, *n* night, *nu* normal unit, *TKV* total kidney volume, *hA* height-adjusted, *TFV* total fibrotic volume

A statistically significant positive linear correlation was observed between length of kidneys and LF nu (*r* = 0.595, *p* < 0.05) and LFd nu (*r* = 0.587, *p* < 0.05). Moreover, a statistically significant positive linear correlation exists between HF nu and TFV (*r* = 0.804, *p* < 0.01) and height-adjusted (ha) TFV (*r* = 0.801, *p* < 0.01). Finally, we found a statistically significant positive linear correlation between HFn nu and TKV (*r* = 0.608, *p* < 0.05), ha-TKV (*r* = 0.685, *p* < 0.01), TFV (*r* = 0.594, *p* < 0.05) and ha-TFV (*r* = 0.615, *p* < 0.05). All linear correlations are showed in Fig. 1.

## Discussion

Autonomic dysfunction is a common finding in patients with chronic kidney disease (CKD) and it is a leading cause of cardiovascular morbidity and mortality. Hyperactivation of the sympathetic system not only leads to an increased basal heart rate, but also promotes myocardial hypertrophy and fibrosis associated with increased risk for sudden cardiac death. HRV is an indirect measure of the sympathovagal interaction at the sinoatrial node and an index of cardiac neural control [7].

In autoimmune diseases characterized by fibrous of the skin and internal organs, a parasympathetic modulation increases in relation to microcirculation dysfunction induced by Raynaud vasospasm [12]. Thus, autonomic system seems to stimulate vasodilatation trough parasympathetic system. Chou et al. [13] have demonstrated that HRV represents a predictor of rapid kidney injury in CKD patients on dialysis. Orscelik et al. [14] showed impaired HRV in 28 ADPKD patients without hypertension, suggesting a link between ADPKD and the autonomic nervous system. Cerasola et al. [15] suggest that increased activity of the sympathetic system could play a role in the pathogenesis of hypertension associated with ADPKD. Hypertension related to ADPKD occurs early and it could be favored by cyst enlargement. This process can cause renal ischemia with renin release, complicated by endothelial dysfunction, reduced NO, and sympathetic tone activation [16]. In ADPKD, continuous RAAS stimulation worsens hypertension and accelerated cyst growth and for this reason, it is not surprising to find in our study that the marker of nightly parasympathetic activity showed a significant positive correlation with TKV and TFV. TKV is a known predictor of CKD progression in ADPKD. In an early stage of kidney disease, TKV and ha-TKV seem to be more accurate markers of disease progression than eGFR [17]. In CKD patients, we usually observe an increased sympathetic activity with lower parasympathetic tone. Some studies showed how HRV in end-stage renal disease (ESRD) leads to an impaired regulation of sinus node activity [7]. In the present study, we can suppose that parasympathetic low activity, mainly during the night, is probably due to the enlargement of kidney volume. This augmentation in TKV could result in kidney fibrosis, induced by chronic hypoxia and vasoconstrictor insults due to the cysts growing. TFV is a non-cystic area of the polycystic kidney, likely characterized by peritubular interstitial fibrosis, tubular dilation, atrophy, and vascular sclerosis [10]. Few studies have evaluated HRV parameters in ADPKD patients to assess their cardiovascular risk, so far [11, 12]. To the best of our knowledge, this is the first study that evaluates TKV and TFV in ADPKD patients in relation to autonomic balance.

The study has some limitations. First, this is a single center study, nonrandomized, with a small cohort of patients since ADPKD is a rare disease. Second, we also have not recorded the onset of cardiovascular events. Anyway, our data show significant results which can address the search to novel early cardiovascular markers in ADPKD population, encumbered by high cardiovascular mortality. Third, the lack of follow-up cannot generalize the results in ADPKD population. Thus, considering the originality as a strength point of this pilot study, in the future, we aim to observe if the progression of renal damage could be correlated to autonomic dysfunction with large number of patients and adequate follow-up. Changes in the MRI parameters and HRV will be evaluated between two measurements (T0 already performed and T1) in terms of their correlations.

It is advisable that future studies in this field will be multicentric and include larger populations possibly more representative of the ADPKD population, to confirm the results. We suppose that the increase in TKV and TFV could lead to a parasympathetic tone hyperactivation, probably in response to hypoxic stress and vasoconstriction due to cystic enlargement.

## Data Availability

The dataset used and analyzed during the current study are available from the corresponding author on reasonable request.
